# Prevalence of oral soft tissue lesions in Vidisha

**DOI:** 10.1186/1756-0500-3-23

**Published:** 2010-01-25

**Authors:** Ravi Mehrotra, Shaji Thomas, Preeti Nair, Shruti Pandya, Mamta Singh, Niraj S Nigam, Pankaj Shukla

**Affiliations:** 1 Department of Pathology, Moti Lal Nehru Medical College, Lowther Road, Allahabad, 211001, India; 2 Departments of Oral and Maxillofacial Surgery, People's College of Dental Sciences & Research Centre, Bhopal, India; 3 Oral Medicine and Radiology, People's College of Dental Sciences & Research Centre, Bhopal, India; 4 Consultant Dental Surgeon, Vidisha, India; 5 District Hospital, Vidisha, India

## Abstract

**Background:**

The purpose of this study was to determine the prevalence of oral soft tissue lesions in patients and to assess their clinicopathological attributes. 3030 subjects belonging to a semi-urban district of Vidisha in Central India were screened. Patients were examined with an overhead examination light and those who were identified with a questionable lesion underwent further investigations. Statistical analysis was done using the SPSS software.

**Findings:**

8.4 percent of the population studied had one or more oral lesions, associated with prosthetic use, trauma and tobacco consumption. With reference to the habit of tobacco use, 635(21%) were smokers, 1272(42%) tobacco chewers, 341(11%) smokers and chewers, while 1464(48%) neither smoked nor chewed. 256 patients were found to have significant mucosal lesions. Of these, 216 cases agreed to undergo scalpel biopsy confirmation. 88 had leukoplakia, 21 had oral submucous fibrosis, 9 showed smoker's melanosis, 6 patients had lichen planus, 17 had dysplasia, 2 patients had squamous cell carcinoma while there was 1 patient each with lichenoid reaction, angina bullosa hemorrhagica, allergic stomatitis and nutritional stomatitis.

**Conclusions:**

The findings in this population reveal a high prevalence of oral soft tissue lesions and a rampant misuse of variety of addictive substances in the community. Close follow up and systematic evaluation is required in this population. There is an urgent need for awareness programs involving the community health workers, dentists and allied medical professionals.

## Background

Oral malignancies are the sixth most common cancer around the globe [1]. Oral mucosal lesions could be due to infection (bacterial, viral, fungal), local trauma and or irritation (traumatic keratosis, irritational fibroma, burns), systemic disease (metabolic or immunological), or related to lifestyle factors such as the usage of tobacco, areca nut, betel quid, or alcohol.

For planning of national or regional oral health promotion programs as well as to prevent and treat oral health problems, baseline data about magnitude of the problem is required. India has a vast geographic area, divided into states, which differ with regard to their socioeconomic, educational, cultural and behavioural traditions. These factors may affect the oral health status. Hence to obtain nationwide representative data, a nationwide study is required. A more practical alternative is to develop regional databases and review data from various regions which may give an understanding of the national scenario.

**Figure 1:**
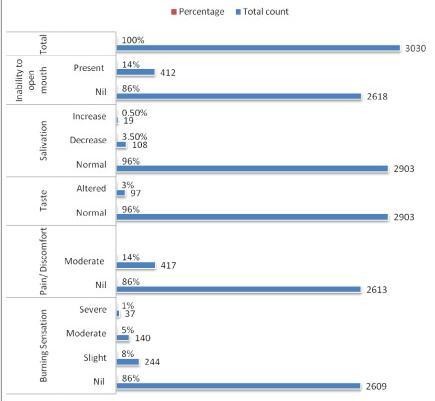
**Distribution of patients according to their complaints**.

In an earlier study, the authors reported that potentially malignant and malignant oral lesions were widespread in the patients visiting a tertiary level referral hospital at Allahabad in North India [2]. This study was undertaken at a semi-urban community at Vidisha in Central India (Fig. 1) to assess magnitude of various oral lesions associated with usage of tobacco, betel nut, betel leaf etc in various forms. As per the 2001 [*update*] Indian census, Vidisha has a population of 1, 25,457. Males constituted 53% of the population and females 47%. The average literacy rate was 70% which was higher than the national average of 59.5%. Male literacy was 77%, and female literacy was 62%. In Vidisha, 15% of the population was under 6 years of age [3]. The vast majority of the people at Vidisha belonged to the lower socio-economic status with poor access to dental care. To the best of our knowledge, there is no data on the oral health status of this community. This study explores the prevalence of oral lesions in this community and attempts to correlate the various risk factors with the lesions found.

## Methods

### Data collection

Individuals presenting to the out-patient department (OPD) of the Government-run District Hospital at Vidisha district in the state of Madhya Pradesh in Central India were screened at an Oral health camp held during the months of May- June 2008, over a period of 10 days by a team of dental and medical specialists. Information about this screening was also disseminated by public announcements, distribution of handbills, media coverage and door-to-door publicity in the remote areas.

### Ethical permission

Permission was obtained from the Institutional ethical committee at Vidisha and written consent was obtained from the participating patients.

### Questionnaire

The WHO Oral Health Assessment Form was used as a basis of a questionnaire and clinical assessment form [4]. General information related to the subjects' oral hygiene practices and habits were collected through interview by paramedical workers. The questionnaire was constructed and administered in English. After a pilot study, the questionnaire was translated into the local language (Hindi) using appropriate and simple words. For validation, the questionnaire was translated back into English. During the survey the questions were read to most of the subjects, as the majority were illiterate.

### Patient Population

Patients who were at least 18 years of age were included in the study. Those who gave a history of usage of tobacco, betel nut and betel leaf in various forms were considered to be at high risk for oral lesions. Prior to the examination, patients rinsed their mouth thoroughly with water and were examined under an incandescent light source. Patients with oral mucosal lesions were identified and lesions that, in the opinion of the investigator warranted histopathological examination, underwent scalpel biopsies.

### Clinical examination

Each patient was evaluated using a pre-designed chart. The clinical diagnosis was established and classified according to the Epidemiology guide for the diagnosis of oral mucosal diseases (WHO) [5]. Correlation, if any, with etiological factors was assessed. The questionnaire included information on general status of the patient, systemic diseases, medications used, age, gender, alcohol and tobacco consumption, habits (trauma) and prosthetic or other appliances use. During the clinical examination the following elements were analyzed: features of the lesion, anatomical location, extension, etiological factors or related factors, dental status, alcohol, tobacco, trauma, use of prosthesis and if these were well adapted. In addition, in those cases requiring further examination, biopsies were performed to establish a definitive diagnosis.

### Statistical analysis

The variables were analyzed on all patients, using the SPSS software (11.0).

## Results

**Figure 2:**
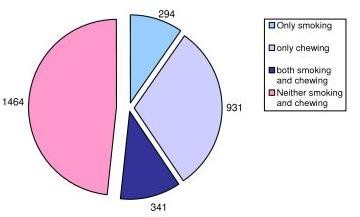
**Distribution of patients according to their personal habits**.

The population under study consisted mainly of individuals living in isolated settlements away from the general population. A total of 3030 subjects were screened. Of these 2150 (71%) were males and 880 (29%) were females. (Fig. 1) Analyzing the clinical symptoms, 417(14%) reported moderate pain/discomfort, 412 (14%) suffered from difficulty in opening the mouth; 244 (8%) patients reported slight burning sensation, 140(5%) reported moderate and 37 (1%) reported severe burning sensation in the oral cavity; 97(3%) patients had altered taste sensation. 19(1%) reported increased while 108(4%) reported decreased salivation. (Fig. 2)

**Figure 3:**
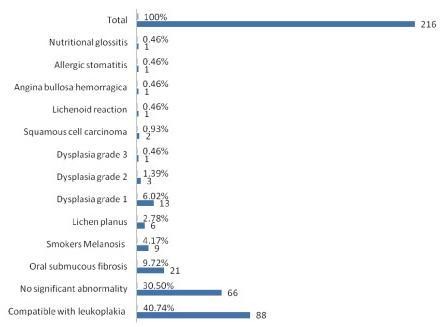
**Histopathological diagnosis of lesions**.

Regarding the habit of tobacco use, 635(21%) were smokers, 1272(42%) tobacco chewers, 341(11%) smokers and chewers, while 1464(48%) neither smoked nor chewed. (Fig. 3) 256 patients were found to have significant mucosal lesions which were maculopapular, erosions, ulcerations or growths. Of these, maximum number of patients i.e. 32(14%) had lesions measuring 1-3 mm. lesion, 8% had lesions of 11-20 mm, while, 27(12%) had lesions more than 20 mm of size.

In 121(53%) patients, both the right and left buccal mucosae were involved. This was followed by involvement of the retromolar trigone. The other areas of the oral cavity like tongue, gingivae, floor of mouth, hard palate, soft palate and alveolar mucosa were less commonly involved.

Of these 256 patients who were identified with abnormalities on clinical examination, 40 patients refused to undergo scalpel biopsy and 216 cases agreed for scalpel biopsy confirmation.

**Figure 4:**
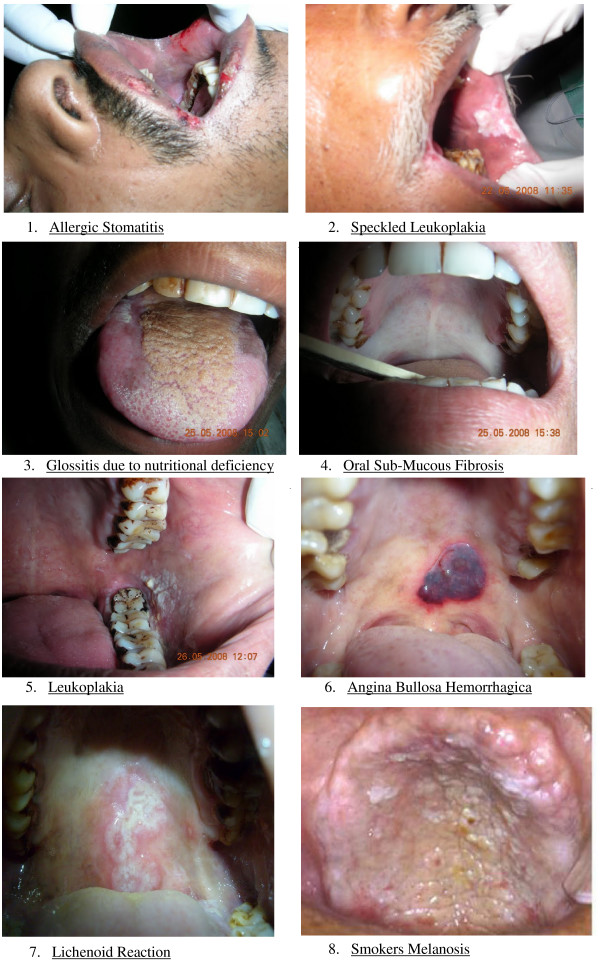
**Spectrum of clinical lesions**.

On histopathological examination, maximum number i.e. 88 (40%) patients had leukoplakia. 17 (11%) patients were found to have dysplasia - of which 13 had dysplasia grade 1, 3 had dysplasia grade II and 1 had dysplasia grade III, while 2 patients were confirmed to have squamous cell carcinoma. Other lesions are detailed in Fig. 4.

On statistical analysis, the patients with leukoplakia had an Odds ratio of 4.5 while chewers had an Odds ratio of 5.6 as compared to non-users. More males had dysplasia than females i.e. 17/18 (p < 0.02) and, at the same time, more males used addictive substances vis-à-vis females (p < 0.02).

The patients with a diagnosis of premalignant conditions like speckled leukoplakia and oral submucous fibrosis were advised abstinence from tobacco and a visit to their dentist, while the patients with dysplasia were advised follow-up at a tertiary level teaching hospital in Bhopal and those with squamous cell carcinoma were advised surgery at the Jawaharlal Cancer Hospital, Bhopal.

## Discussion

Only limited information on oral mucosal abnormalities in the rural or semi-urban population of India is available, however few isolated studies of prevalent lesions have been reported [6-11]. The prevalence of oral lesions in population has been documented in other parts of the world like Colombia [12], Mexico [13], Brazil [14], Chile [15], Spain [16], Argentina [17], USA[18], Israel [19] and Cambodia [20], mainly based on clinical evaluation of the lesions. In contrast, Correa et al [21] and Dehler et al [22] conducted prevalence studies based on the clinicopathological correlation, evaluating the biopsies of the observed lesions.

While emerging lifestyle and food habits have been contributing factors, the problem of bad oral health is compounded by a low dentist to population ratio. The World Health Organisation (WHO) recommends a 1: 7500 dentist to population ratio whereas the dentist to population ratio in India is as low as 1:22500 [23]. In 2004, India had one dentist for 10,000 persons in urban areas and about 2.5 lakh persons in rural areas. Almost three-fourths of the total number of dentists were clustered in urban areas, which house only one-fourth of the country's population [24]. This limits the curative approach to tackle dental problems in rural areas while it is widely acknowledged that oral cancer can best be prevented through early detection and primary prevention. Unfortunately, the awareness levels of lesions associated with usage of addictive agents continue to remain abysmally low.

This study was a community survey, in which the prevalence of clinically significant oral lesions was 8.4% - which was higher in comparison to a previous study from Chennai (4.1%). This could probably be due to higher prevalence of smoking and/or tobacco chewing (52%) in this study in comparison to 31% reported by Saraswathi et al [6]. Vellappally et al found that in a survey of 805 subjects for dental caries, the highest prevalence of oral mucosal lesions were present in tobacco chewers (22.7%) followed by regular smokers (12.9%), occasional smokers (8.6%), ex-smokers (5.1%) and non tobacco users (2.8%) [11]. The prevalence figure of oral lesions was 8.4% covering all age groups. On the other hand, Gonzalez et al [13] in Mexico, demonstrated a prevalence of 23.2% in the elderly. Sanchez reporting in Spain, documented 39% of aged patients had oral mucosa alterations [16].

Of the clinically significant lesions which were biopsied, the percentage of patients suffering from leukoplakia was 40.7%, oral submucous fibrosis 9.7% and lichen planus 2.7% which was higher to those found by Saraswathi et al (0.59%, 0.55% and 0.15% respectively) Prevalence of smoker's melanosis was 2.3% in this study while it was lesser in Chennai (1.14%) [6]. Dysplasia was found in 17 patients out of which 6% was found with grade I, 1.4% with grade II and 0.5% with grade III while squamous cell carcinoma was found in 0.93% in this study. These findings reveal higher percentages than similar studies from India, [6-11] and this difference may probably be explained by the fact that, unlike most other clinical studies, in this report, histopathological confirmation was obtained in most of the cases.

Subjects who smoked or chewed tobacco in any form had a far higher incidence of oral lesions vis-à-vis non-users. On assessing the correlation of habits with incidence of leukoplakia, smokers were found to have an Odds ratio of 4.5 while chewers had 5.6 as compared to non-users. This was less than findings of Saraswathi et al who reported figures of 5.08 and 6.82, respectively. Subjects who chewed areca nut with or without tobacco had a higher prevalence of oral submucous fibrosis, similar to earlier findings[6].

Since the information on habits was garnered by the patients or attendants by a questionnaire, the possibility of an information bias should be considered while interpreting the results. Another limitation of the study was that due to the rather small sample size, inherent in a population survey vis-à-vis a hospital survey, there is a lack of generalizability and limited statistical significance.

## Conclusion

This survey high-lighted the rampant misuse of variety of addictive substances as well as the high prevalence of oral lesions in the community. There is an urgent need for awareness programs utilizing the community health workers, dentists and allied medical professionals. It is hoped that these results will form the basis of a state level, followed by a national level survey of oral lesions.

## Competing interests

The authors declare that they have no competing interests.

## Authors' contributions

RM, SJ and PJ carried out survey, analysis and drafted the manuscript. SP, MS, NSN and PS conceived of the study, participated in its design and coordination as well as helped to draft the manuscript.
